# Voting Restrictions and Increased Odds of Adverse Birth Outcomes in the US

**DOI:** 10.1007/s40615-024-02253-0

**Published:** 2024-12-06

**Authors:** Sze Yan Liu, Erin Grinshteyn, Daniel Cook, Roman Pabayo

**Affiliations:** 1 Department of Public Health, College for Community Health, Montclair State University, University Hall, Room 4153, 1 Normal Avenue, Montclair, NJ 07043 USA; 2 School of Nursing and Health Professions, University of San Francisco, San Francisco, USA; 3 School of Public Health, University of Nevada-Reno, Reno, USA; 4 School of Public Health, University of Alberta, Edmonton, Canada

**Keywords:** Preterm births, Small-for-gestational age, Racial inequity, Structural racism, Voting restrictions

## Abstract

**Background:**

Disparities persist in adverse birth outcomes — preterm birth and small-for-gestational age (SGA) among racialized populations. Previous studies have indicated that voting restrictions are associated with health outcomes, such as access to health insurance and teenage birth rates. This paper examines whether the association between voting restrictions and adverse birth outcomes varies according to birthing individuals’ race/ethnicity.

**Methods:**

These analyses merged individual-level 2019–2020 Pregnancy Risk Assessment Monitoring System (PRAMS, 8th edition) data with state-level exposure information. The exposure, the Cost of Voting Index (COVI), is a 2020 state-level measure of voting restrictions, and the outcomes were preterm birth and SGA. Multilevel logistic regression, survey-weighted models adjusted for sociodemographic and geographically-based characteristics. Subanalyses examined if the association differed by race (non-Hispanic White, non-Hispanic Black, Hispanic, API, Other).

**Results:**

In the unadjusted model, a standard deviation increase in COVI was associated with increased odds of preterm birth (OR = 1.11, 95% CI = 0.98, 1.25) and SGA (OR = 1.12, 95% CI = 1.02, 1.22). The association for SGA was still significant in the fully adjusted models. Results differed by race/ethnicity with the largest effects among API (OR = 1.20, 95% CI = 0.95, 1.52) for preterm birth and OR = 1.27, 95% CI = 1.01, 1.59) for SGA respectively).

**Conclusion:**

Our results suggest structural voting barriers disproportionately increase the odds of adverse birth outcomes, especially for API-birthing individuals. Increasing voting restrictions may amplify existing birth inequities.

## Background

Adverse birth outcomes, which include preterm birth (PTB) and being born small for gestational age (SGA), are serious national and global public health concerns [1]. In the US, one in 10 babies is born prematurely (i.e., less than 37 weeks), and one in 10 infants is SGA (i.e., birth weight below the 10th percentile for gestational age-specific birth weight reference). Babies born with any of the above conditions are at higher risk of poor infant health outcomes and infant death [2–4].

In addition, racial/ethnic inequities in birth outcomes are persistent and are a cause of concern. Non-Hispanic black women experience the highest rates of adverse birth outcomes among all racial groups in the US. For example, non-Hispanic black women are two times more likely to have a preterm birth compared with non-Hispanic white women [5]. Preterm birth is also higher among babies born to American Indian women [6, 7]. Some studies have shown that babies born to mothers of varying Hispanic subgroups were more likely to be small-for-gestational age compared to babies born to white mothers [8, 9]. Individual-level characteristics such as differences in income or health behaviors cannot fully explain racial inequities in adverse birth outcomes [10, 11].

Infants born with PTB or SGA are also at higher risk of adverse health outcomes throughout their childhood, particularly for neurological disorders and developmental delays [12]. For example, SGA is associated with lower cognitive scores during childhood [13, 14]. According to one study, PTB accounts for an estimated 55% of cerebral palsy and 10–20% of other developmental delays [15]. As a result of these short and long-term health consequences, the impact of these adverse birth outcomes extends to increased healthcare expenditure and decreased human capital [16–18].

Previous research on adverse birth outcomes has primarily focused on individual-level risk factors. While health characteristics of birthing individuals — such as chronic stress, hypertension, smoking, and undernutrition — are well-known risk factors [19–21]. Racial disparities in adverse birth outcomes are also widely documented. According to previous studies, the highest risk of LBW in the USA is observed for non-Hispanic Black birthing people, followed by API individuals compared with those who are non-Hispanic White people [22–24]. Similarly, babies of Asian descent are most likely to have the lowest fetal weight compared with babies of other races/ethnicities [25]. However, individual-level characteristics cannot fully account for these inequities in poor birth outcomes [26]. In addition, individual-level risk factors, such as race, are indicators for larger, contextual characteristics. Little is known about modifiable, contextual risk factors for adverse birth outcomes.

It is crucial to understand that states in the USA wield substantial policy powers that shape social determinants, directly impacting health inequities. For example, a wide variation in state policies affecting employment, education, housing, the environment, and healthcare access is associated with considerable geographical variations in health and morbidity [27–29]. As an upstream determinant of state policies, voting restrictions may affect population health.

The voter suppression framework describes how voter suppression in the USA inequitably impacts people of color and, therefore, is a form of structural racism [30–32]. Voting barriers lead to worse health outcomes among people of color because this structural exclusion may decrease access to resources and social capital [33]. Elected officials are more likely to respond to the preferences of voters than nonvoters [34]. Therefore, voting barriers may contribute to health inequities if elected officials support social and health policies that differentially ignore nonvoters’ needs and preferences. In addition, the voter suppression framework describes not being able to vote may adversely affect health through feelings of disempowerment and social exclusion, which may further affect mental health and overall well-being [33]. It is in these ways that voting restrictions operate as a form of structural racism that further reinforces other fundamental causes of health inequities.

While a growing body of research has linked voting restrictions to various area-level population health outcomes, such as teenage birth rates, COVID mortality and morbidity rates, and individual health outcomes like insurance status [35–38], a significant gap remains. No study to date has explored the potential impact of voting barriers on infant health, making this an uncharted area of investigation.

This paper aims to address this gap in the literature. We add to the nascent literature studying the impact of voting restrictions by examining its effect on infants. Given the emerging evidence that voting restrictions have an impact on adults’ healthcare access and health, we hypothesize that voting restrictions will spill over and affect infants’ health. Furthermore, we examine whether race/ethnicity modifies the association between voting barriers and birth outcomes.

## Methods

### Sample

This study used individual-level 2019–2020 Pregnancy Risk Assessment Monitoring System (PRAMS, 8th edition) data merged with state-level information from the USA Census. PRAMS is an annual population-based survey of self-reported sociodemographic risk factors, and behaviors and experiences before, during, and after pregnancy in the US. PRAMS is conducted by the Centers for Disease Control and Prevention’s Division of Reproductive Health in collaboration with individual state health departments. Participants are sampled from state birth certificates [39]. PRAMS participants complete the survey 2 to 6 months after giving birth. All sites administer a standard set of PRAMS questions and may choose to include optional modules of questions. More details about PRAMS are available on the PRAMS website (https://www.cdc.gov/prams/methodology.htm). Our 2019–2020 national PRAMS dataset was missing data from California, Idaho, Indiana, Nevada, New York, Ohio, Oklahoma, South Carolina, or Texas because those states required additional state approval at the time of this study. This study protocol was reviewed by the Montclair State University Institutional Review Board and considered exempt from human subjects research.

### Exposure

We utilized a state-level measure of voting barriers, the Cost of Voting Index (COVI), developed by political scientists [40]. The COVI measure is a variable that reflects the presence of various state voting restriction laws. These laws, such as the disallowance of early voting, the requirement of specific types of photo IDs, and the restriction of polling hours, are common state-passed voting restrictions. A higher COVI score indicates a greater number of barriers and more voter suppression. We applied a *Z*-transformation, a statistical process that standardizes the score for easier interpretation. Our model results can be interpreted as the change in odds of the infant birth outcome associated with one standard deviation increase in the COVI score or increased voter suppression. For this study, we specifically used the 2020 COVI scores because they correspond most directly with the 2019–2020 PRAMS data. Using an older COVI score is problematic because we feel four years before the individual gave birth is outside of the exposure time period of interest.

### Outcome

We examined two common adverse birth outcomes — SGA and PTB. We used the standard definition of premature birth as birth before 37 weeks [41] and SGA as having a birth weight below the 10th percentile for gestational age-specific birth weight reference [42]. These outcomes were derived from birth certificate data included in PRAMS.

### Covariates

We included sociodemographic characteristics strongly associated with adverse birth outcomes including age, marital status, maternal educational attainment, and income in relation to the federal poverty level. We calculated the federal poverty level (FPL) by randomly assigning a number in the self-reported income category to each respondent and accounted for the number of dependents, a method used and described in previous studies [43]. Our federal poverty-level variable was ordinal: 0–100% FPL, 101–200% FPL, 201% and greater FPL. We also included maternal race/ethnicity, created as a nominal variable with the categories of non-Hispanic White, non-Hispanic Black, Hispanic, non-Hispanic Asian/Pacific Islander, non-Hispanic Other (American Indian, Alaskan Native, and mixed race), and missing maternal race/ethnicity. Maternal age was also included as an ordinal variable with five categories (i.e., less than 20, 20–24, 25–29, 30–34, 35 and older). Educational attainment was an ordinal variable consisting of four categories of years of schooling, from 0 to 11, 12 years, 12 to 15, and 16 or more years of schooling. Pre-pregnancy health insurance status and health insurance during pregnancy were included in the adjusted models as nominal variables consisting of five categories each — no health insurance, public (Medicaid/Medicare/other government plan), private health insurance, other health insurance not specified, and unknown. Marital status and parity were coded as binary variables (i.e., married/not married and nulliparous/not, respectively). We also included a variable indicating whether the participant lived in an urban or rural area. Finally, we included variables related to previous pregnancies — parity and having a preterm birth before this birth.

Our fully adjusted model also took into account geographically based characteristics, which play a significant role in understanding the differences in state-level incidence of adverse birth outcomes. These characteristics include state-level population size and median household income, as highlighted in previous studies [44, 45]. We used annual state-based covariate data from 2019 to 2020, the same years as the PRAMS data because it corresponds to our exposure time window.

### Analysis

We conducted a multilevel logistic regression analysis to account for the clustering of participants within the state-specific sampling geographic strata. Given the well-documented racial disparities in birth outcomes, we decided a priori to test whether the association between voting barriers and adverse birth outcomes differed by race/ethnicity using an interaction term. Given the statistical significance of the interaction term in the fully adjusted model, we then proceeded to conduct analyses stratified by race/ethnicity. We rescaled the PRAMS survey weights so the new weights would sum to the effective cluster size which, in this dataset, is the state-specific sampling geographic strata. This rescaling strategy is recommended for use in multilevel models [46]. We included these rescaled weights in all analyses to account for survey sampling techniques. We used Stata 18.0 for all analyses.

## Results

There were 80,691 birthing individuals in our sample with complete information on the outcome and covariates (94%). Table 1 provides a detailed breakdown of the sociodemographic characteristics of the sample. Over 50% of the participants were 30 and older at birth, and over 70% resided in urban areas. The participants were highly educated, with a significant 63% reporting more than 12 years of education. Moreover, approximately 79% of the participants were insured before the pregnancy, and 84% had health insurance during their pregnancy. In addition, most delivered a singleton birth (96.7%).

**Table 1:** Sociodemographic characteristics of the sample, PRAMS (*n* = 80,691)

	Total (%)
Sociodemographic characteristics of the individuals giving birth	
Age	
Less than 20 years	3381 (4%)
20–24 years	14,182 (18%)
25–29 years	23,053 (29%)
30–34 years	24,334 (30%)
35 and older	15,726 (20%)
Education	
Less than 12 years	9139 (11%)
12 years	19,568 (24%)
13–15 years	22,976 (28%)
16 years of more	28,352 (35%)
Unknown	656 (< 1%)
Maternal race/ethnicity	
Non-Hispanic White	36,532 (45%)
Non-Hispanic Black	14,264 (18%)
Hispanic	13,272 (16%)
Asian Pacific Islander	5790 (7%)
Multiracial/Other	8363 (10%)
Don’t know	2470 (3%)
Marital status	
Married/partnered	47,851 (59%)
Single/divorced/widowed	32,778 (41%)
Urban/rural	
Urban	62,431 (77%)
Rural	18,228 (23%)
Unknown	32 (< 1%)
Federal poverty level	
0–100% FPL	19,239 (24%)
101–200% FPL	14,699 (18%)
> 200% FPL	38,604 (48%)
Missing/unknown	8149 (10%)
Healthcare access and health characteristics	
Health insurance during pregnancy	
None	1777 (2%)
Private	36,044 (45%)
Public	30,208 (37%)
Other	1600 (2%)
Unknown	11,062 (14%)
Health insurance before pregnancy	
None	9662 (12%)
Private	42,068 (52%)
Public	21,037 (26%)
Other	1768 (2%)
Unknown	6156 (8%)
Previous preterm birth	
Yes	3938 (5%)
No	76,645 (95%)
Unknown/DK	108 (< 1%)
Birth characteristics	
Gender of infant	
Male	40,631 (50%)
Female	40,047 (50%)
Plurality	
One	76,523 (95%)
Two	2561 (3%)
Three or more	1607 (2%)
Year of birth	
2019	41,653 (52%)
2020	39,029 (48%)

Results from our multilevel models are shown in Table 2. In the unadjusted model, a standard deviation increase in COVI was associated with increased odds of preterm birth (OR = 1.11, 95% CI = 1.01, 1.29), and small for gestational age (OR = 1.11, 95% CI = 1.02, 1.20) outcomes. While the associations for preterm birth were no longer statistically significant once we included potential individual and state-level potential confounders, the measure of association remained unchanged (adjusted OR = 1.11, 95% CI = 0.98, 1.25). Other characteristics in the adjusted model which were also associated with a statistically significant increased odds for preterm birth included older maternal age, not being currently married, infant gender, and being part of a multiple birth vs. singleton birth (Table 2).

**Table 2:** Odds ratios* associated with a one standard deviation increase in COVI, PRAMS 2019–2020

	Preterm birth		SGA	
	Unadjusted	Adjusted	Unadjusted	Adjusted
*Z*-score COVI	1.14 (1.01, 1.29)	1.11 (0.92, 1.34)	1.11 (1.02, 1.20)	1.12 (1.00, 1.25)
Maternal race/ethnicity				
Non-Hispanic White	Reference		Reference	
Non-Hispanic Black	–	1.05 (0.96, 1.14)	–	0.68 (0.62, 0.74)
Hispanic	–	1.02 (0.93, 1.12)	–	0.91 (0.84, 0.99)
API	–	0.88 (0.78, 1.01)	–	1.02 (0.90, 1.14)
Other	–	0.87 (0.77, 0.99)	–	0.86 (0.77, 0.96)
Missing	–	0.90 (0.70, 1.15)	–	0.84 (0.67, 1.05)
Maternal education				
0 to 11 years	–	0.97 (0.89, 1.05)	–	1.15 (1.06, 1.24)
12 years	Reference		Reference	
13 to 15 years	–	0.95 (0.89, 1.02)	–	0.87 (0.82, 0.93)
> = 16 years	–	0.85 (0.78, 0.92)	–	0.83 (0.77, 0.90)
Missing	–	1.08 (0.83, 1.40)	–	1.20 (0.94, 1.53)
Maternal age				
Less than 20 years	–	1.00 (0.88, 1.13)	–	0.86 (0.76, 0.96)
20–24 years	Reference		Reference	
25–29 years	–	1.08 (1.00, 1.16)	–	1.15 (1.07, 1.24)
30–34 years	–	1.28 (1.18, 1.38)	–	1.13 (1.04, 1.21)
35 and older	–	1.54 (1.41, 1.68)	–	1.22 (1.12, 1.33)
Missing	–	–	–	2.45 (0.27, 22.57)
Marital status				
Currently married	Reference		Reference	
Currently not married	–	1.07 (1.01, 1.14)	–	1.12 (1.05, 1.18)
Missing	–	2.55 (1.20, 5.43)	–	0.90 (0.42, 1.98)
Current health insurance				
None	Reference		Reference	
Private	–	1.05 (0.95, 1.17)	–	0.92 (0.83, 1.02)
Public	–	1.12 (1.02, 1.22)	–	1.04 (0.95, 1.13)
Other	–	0.86 (0.71, 1.04)	–	0.90 (0.75, 1.07)
Missing	–	1.07 (0.95, 1.20)	–	0.98 (0.87, 1.09)
Health Insurance during pregnancy				
None	Reference		Reference	
Private	–	0.93 (0.77, 1.12)	–	1.22 (0.97, 1.34)
Public	–	0.86 (0.73, 1.03)	–	1.07 (0.99, 1.16)
Other	–	0.93 (0.73, 1.19)	–	0.99 (0.91, 1.07)
Missing	–	0.95 (0.79, 1.14)	–	1.18 (1.07, 1.30)
Household poverty level				
Less than 100%	–	1.07 (0.98, 1.17)	–	1.23 (1.13, 1.34)
100–199%	–	1.03 (0.95, 1.13)	–	1.07 (0.99, 1.16)
200–399%	Reference		Reference	
> = 400%	–	0.92 (0.84, 1.00)	–	0.99 (0.91, 1.07)
Missing	–	1.02 (0.92, 1.13)	–	1.18 (1.07, 1.30)
Year of birth				
2019	Reference		Reference	
2020	–	1.08 (0.75, 1.56)	–	1.03 (0.83, 1.29)
Infant gender				
Male	Reference		Reference	
Female	–	0.73 (0.70, 0.77)	–	1.03 (0.99, 1.08)
Missing	–	0.32 (0.04, 2.67)	–	
Singleton birth				
Yes	Reference		Reference	
No	–	7.84 (6.99, 8.79)	–	–
Missing	–	1.68 (0.38, 7.40)	–	1.94 (0.75, 4.82)
Previous birth				
None	Reference		Reference	
One	–	0.77 (0.73, 0.82)	–	0.65 (0.61, 0.68)
Two or more	–	0.75 (0.70, 0.81)	–	0.54 (0.51, 0.58)
Missing	–	1.30 (0.73, 2.31)	–	0.66 (0.35, 1.23)
Previous preterm birth				
Yes	–	4.10 (3.75, 4.49)	–	0.79 (0.71, 0.88)
No	Reference		Reference	
Missing	–	0.90 (0.46, 1.76)	–	1.04 (0.52, 2.06)
Urbanicity				
Urban	Reference		Reference	
Rural	–	0.96 (0.90, 1.03)	–	1.03 (0.97, 1.10)
Missing	–	1.89 (0.52, 6.94)	–	1.22 (0.39, 3.80)
State-level variables				
*Z*-score total population	–	0.92 (0.66, 1.28)	–	1.00 (0.82, 1.23)
*Z*-score median household income	–	0.94 (0.77, 1.14)	–	1.02 (0.91, 1.15)

^*^Adjusted for individual-level characteristics and state-level characteristics, multilevel model clustered on the stratum-level

For SGA, the adjusted model found that a one standard deviation increase in COVI was still associated with a statistically significant increase in odds of SGA (OR = 1.12, 95% CI = 1.02, 1.22). Higher education level, previous birth, and having a previous preterm birth were also associated with lower odds of SGA. By contrast, older age, not being currently married, being non-Hispanic Black, Black, or Other were associated with lower odds of SGA compared to non-Hispanic White.

**Fig. 1:**
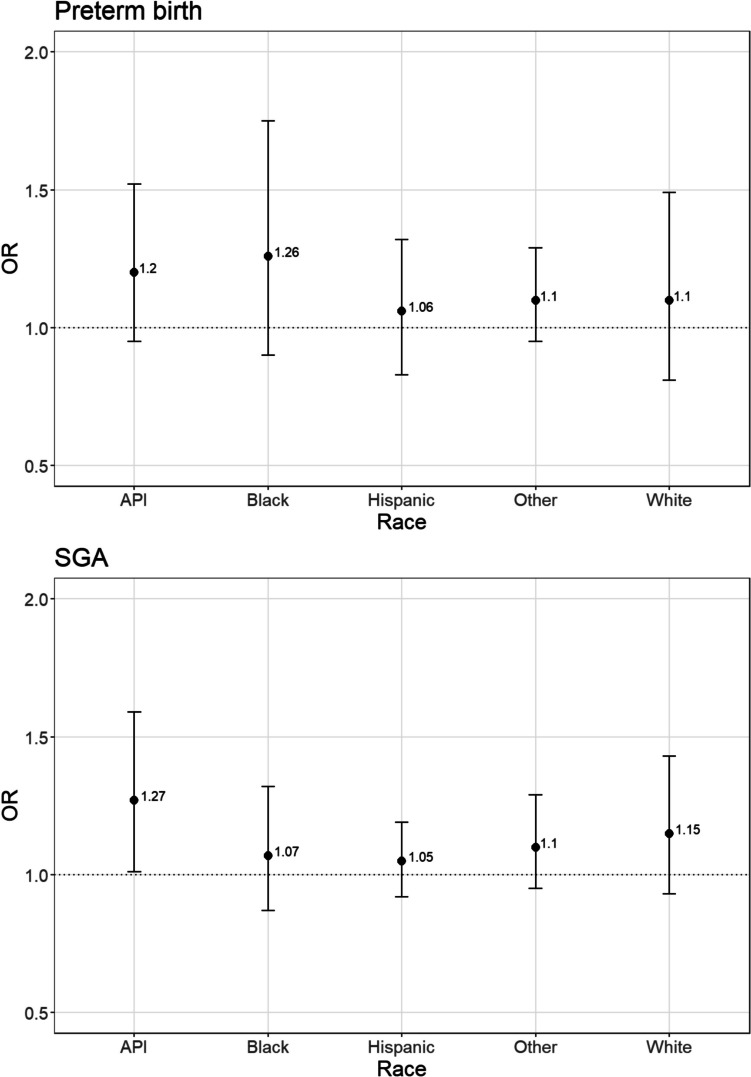
Adjusted odds ratios for preterm birth and SGA associated with a one standard deviation increase in COVI, PRAMS 2019–2020

However, our analyses indicate heterogeneity in the association between voting barriers and adverse birth outcomes. While one standard deviation in state-level voting barriers was associated with an increase in odds of adverse birth outcomes in the race-stratified analyses, the model estimates were not always statistically significant. Among non-Hispanic white birthing individuals, a standard deviation increase in voting barriers was not associated with statistically significant odds of any adverse birth outcomes (Fig. 1). By contrast, a one standard deviation increase in state-level voting barriers was associated with increased odds of SGA (OR = 1.27, 95% CI = 1.01, 1.59) among API-birthing individuals. In the API-stratified analysis, the *E*-value was 1.86 for SGA. These calculated *E*-values suggest an unmeasured confounder that could explain our observed odds ratio would have to be associated with the exposure and the outcome by a minimum odds ratio of this calculated *E*-value. We also found a one standard deviation increase in state-level voting barriers was associated with increased odds of preterm birth among non-Hispanic Black birthing individuals (OR = 1.26, 95% CI = 0.90, 1.75) although it was not statistically significant.

## Discussion

Our results indicate an increase in state-level voting barriers was associated with an increase in odds of preterm birth and SGA. In addition, we found these associations to differ by race/ethnicity. In our race-stratified analyses, we found the largest association between one standard deviation increase in voting barriers and preterm birth and SGA among API-birthing individuals. An increase in voting barriers was also associated with an increase in odds of preterm birth among Black birthing individuals (OR = 1.27, 95% CI = 0.90, 1.75), but this increase was not quite statistically significant.

In general, our results are similar to those of previous studies. For example, our findings that older age, educational level, and specific racial/ethnic groups are associated with higher odds of SGA are similar to those of previous studies [47]. However, our main finding for racial/ethnic differences in the association between voting barriers and SGA had both similarities and differences from previous studies that examined voting barriers. A previous study using BRFSS data found that an increase in voting barriers was associated with an increase in odds of being uninsured among Black, Hispanic, and API 18–64-year-old Americans, with the largest association noted among API [48]. Unlike that earlier study examining health insurance status, we did not find a statistically significant increase in odds of adverse birth outcomes among Blacks and Hispanics. This may be because three states with a large Black population (i.e., New York, Texas, and California) chose not to include their data in the overall PRAMS dataset, which may have underpowered our race-specific subanalyses. In addition, there may be measurement errors associated with using the cost of voting index to capture the structural racism created by voting barriers among Blacks. Black birthing individuals’ lived experiences of structural, institutional, interpersonal, and internalized racism are complex. Previous studies examining the effects of racism and adverse birth outcomes among Black birthing individuals have been mixed, in part, reflecting the varied conceptualization and measurement of API’s lived experiences of racism/racial discrimination [49–51].

Similar to the previous study looking at health insurance status [48], we found the largest association between voting suppression and adverse birth outcomes was among the API subsample. Researchers in the Asian American field have long noted that API individuals face multiple structural voting barriers in the USA [52–55]. API may be especially sensitive to recent voter suppression factors because of their cultural history in the US. Over 30% of APIs in the USA have limited English skills [56], and 68% of API adults are immigrants [57], which may make changes in voting requirements challenging. Over 66% of API voters, a larger percentage than any other racial/ethnicity, use early and mail-in voting [58], so restrictions explicitly targeting these areas will disproportionately affect API voters. Similar to Blacks, APIs are also 20% less likely than Whites to have forms of identification, an increasingly common voting requirement [59]. Qualitative studies also support the adverse lived experiences of API affected by voting restrictions [54]. For these reasons, APIs may be especially vulnerable to voting suppression.

State-level legislation is increasingly recognized as a source of potential intervention to impact population health and decrease health inequities [27]. The growing body of research demonstrating a strong association between state-level policies and adverse birth outcomes has focused on policies that are directly related to birthing people’s healthcare access, such as Medicaid expansion [60] and reproductive rights [61]. Our results suggest that reducing structural racism, in the form of removing voting barriers, could improve birth outcomes for people of color, presumably through having political representation supporting their needs. Voting restrictions may lead to specific racial/ethnic groups having less representation, e.g., representatives who can act on their health interests, which will have a negative impact on health inequity. Further research is needed to examine the various possible pathways connecting voting restrictions and adverse birth outcomes among people of color. For example, voting restrictions were significantly associated with decreased odds of access to health insurance, particularly among API populations (reference our paper). Decreased access to health insurance is a risk factor for adverse birth outcomes [62]. Perhaps, reduced access to health insurance and, thereby, health services utilization is on the causal pathway between voter restrictions and adverse birth outcomes among people of color.

Our study has several study limitations. For example, associations cannot be interpreted as causal. In addition, there may be residual confounding from covariates which were not measured in the PRAMS dataset. For example, the PRAMS dataset does not contain information on maternal place of birth, a known contributor to racial differences in birth outcomes. However, the *E*-values of our stratified subanalyses indicate any residual confounder would need to be strongly associated with the exposure and the outcome to completely explain the measure of association that we found in this study. Our study sample also did not include the births from several large states such as California, New York, and Texas because these states opted to not include their data in the publicly available dataset from CDC. This may limit the generalizability of our results to smaller, less populated states. The absence of these large, racially diverse states in our analytical sample may lead to less variation in our COVI exposure in our sample which may reduce the statistical significance of our results. In addition, the lack of data on these three states may also explain why we do not find the association between voting barriers and infant outcomes to differ significantly by race in our study. Further studies with information from all fifty states are needed to test. Finally, our study assumes voting restrictions have an imminent effect on birth outcomes and does not account for historical restrictions that may have lagged effects on birth outcomes.

Despite these limitations, our study addresses a critical gap in the literature. A limited number of studies specifically examine voting barriers and health outcomes. While all the studies have found voting suppression associated with worse health outcomes, most of these published studies are ecological [36, 63, 64]. These ecological studies cannot examine whether the effects of voting suppression on health differ by individual-level characteristics. Furthermore, no previous study examined the association between voting barriers and infant/children’s health. It is important to understand whether current policies may potentially affect future generations.

Despite the growing racial diversity in the US, the majority of the state legislatures, county officials, and other elected politicians remain white males [65]. Voting restrictions may be contributing to this inequitable political representation of our country’s demographics. Furthermore, such restrictions may directly limit the ability of specific subpopulations to vote for representatives who can advocate for their health interests and the interests of their infants. Our study results support the greater argument that we may need to increase the political capacity of subpopulations who are disadvantaged to address health inequity [66]. Reducing birth inequities will require addressing upstream structural barriers such as voting restrictions.

## Data Availability

PRAMS data is publicly available upon request from CDC.

## Code Availability

Code is available upon request from SYL.
